# Pandemic paradox: the impact of the COVID-19 on the global and Brazilian tuberculosis epidemics

**DOI:** 10.3389/fpubh.2024.1399860

**Published:** 2024-07-26

**Authors:** Eloise T. M. Filardi, Manuela B. Pucca, João Pessoa Araujo Junior, Paulo I. da Costa

**Affiliations:** ^1^Department of Clinical Analysis, School of Pharmaceutical Sciences, São Paulo State University (UNESP), Araraquara, São Paulo, Brazil; ^2^Department of Chemical and Biological Sciences, Institute of Biosciences, São Paulo State University (UNESP), Botucatu, São Paulo, Brazil

**Keywords:** SARS-CoV-2, tuberculosis, society impact, sanitary measures, pandemic, epidemic, public health

Throughout the nearly 3-year period of the SARS-CoV-2 pandemic, various strategies were implemented to contain the virus, including the use of masks, frequent hand washing, social distancing, and quarantine. This period represented an extraordinary threat to public health, impacting populations due to the severity of virus infection, often requiring hospitalization and intubation, with patients staying in intensive care units (ICUs) (1). Alongside these events, there were implications for mental health, characterized by a significant increase in reports of anxiety, depression, distress, sleep disorders, and post-traumatic stress during periods of confinement and social isolation. Furthermore, the repercussions of the pandemic extended beyond the health sector, manifesting in notable effects on economic, food, educational, and tourism domains (2).

The trajectory of the COVID-19 pandemic exerted substantial influence on the Tuberculosis (TB) epidemic, manifesting in a notable decrease in notifications at the peak of the pandemic in 2020. The number of new TB cases plummeted from 7.1 million in 2019 to 5.8 million in 2020, marking an approximate 18% decline in official TB notifications. As 2021 unfolded, numerous countries achieved significant strides in vaccination efforts, contributing to a lessening of the severity of COVID-19 cases and a reduction in the mortality rate associated with SARS-CoV-2. This positive trend facilitated a gradual normalization in the functioning of healthcare sectors, even amid substantial global spikes in COVID-19 cases. The severity of illness among SARS-CoV-2 carriers became less pronounced compared to the early stages of the pandemic, alleviating the strain on healthcare systems, and enabling a return to normalcy in healthcare services. This improvement was evident in the resumption of notifications for other diseases, including tuberculosis, which saw ~6.4 million new cases in 2021 (Figure 1A).

**Figure 1:**
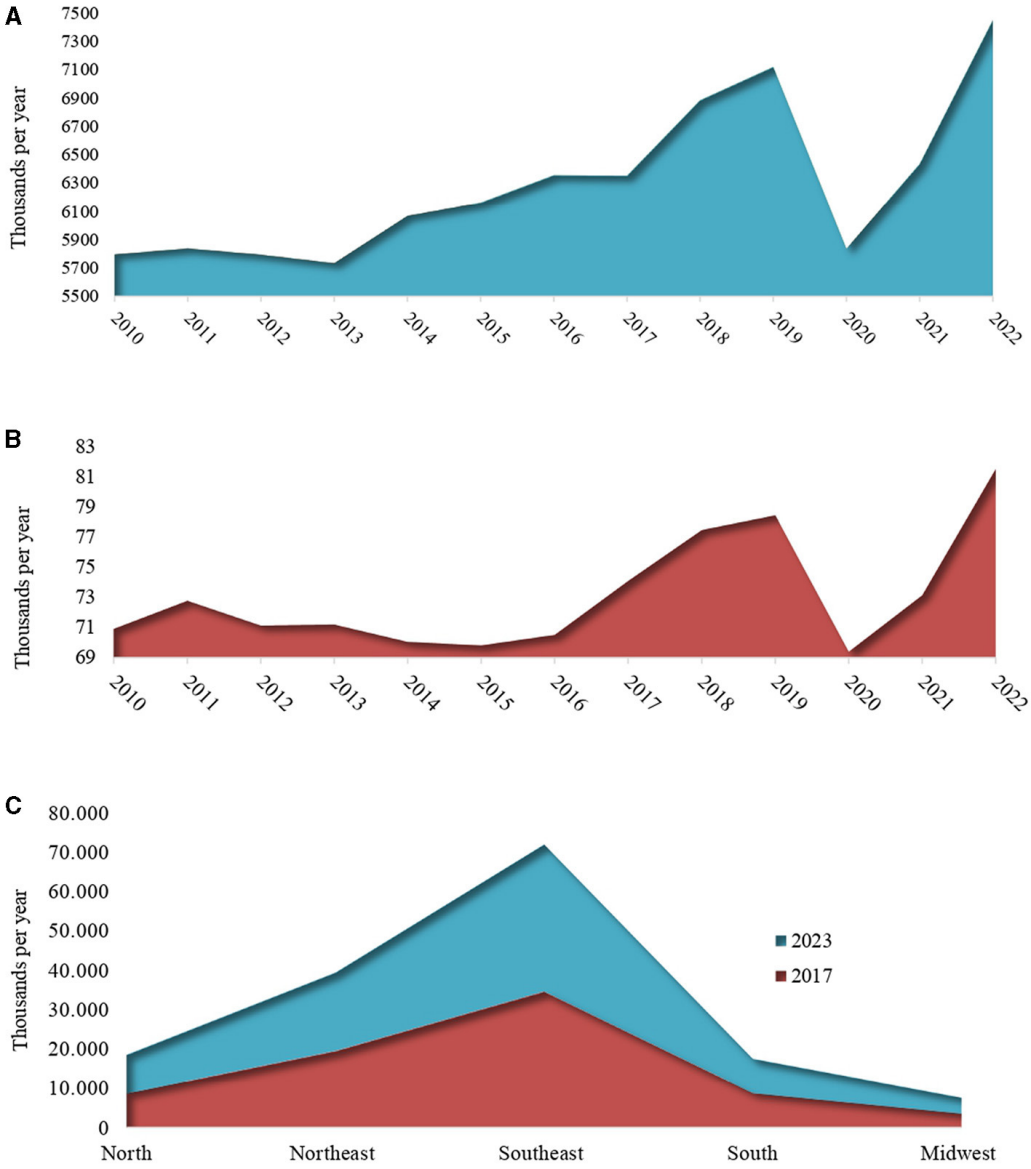
Trends in the estimated number of incident new tuberculosis cases globally **(A)**, in Brazil **(B)**, and epidemiological indicators of new tuberculosis cases by Federative Units of Brazil **(C)**. Data source: WHO Global Tuberculosis Report, 2023 and the Epidemiological Bulletin (Brazil), 2017 and 2023 (3, 4).

Despite the widespread use of masks and the implementation of various sanitary strategies, aimed not only at combating the COVID-19 virus but also other airborne pathogens, the incidence of tuberculosis cases increased substantially in 2022. Official notifications reported a staggering 7.5 million newly diagnosed individuals with TB, surpassing the 2019 figure of 7.1 million by 4.5% (Figure 1A). This paradoxical rise occurred despite 2 years of stringent sanitary measures, with people remaining apprehensive about venturing outside and predominantly confined to their homes.

In Brazil, from 2015 to 2022, the tuberculosis incidence rate also showed a similar trend with a 12.1% reduction in tuberculosis new cases from 2019 to 2020. On the other hand, in 2021, the incidence rate resumed its upward trajectory, reaching 34.9 cases per 100,000 inhabitants (74,385 new cases). This ascending trend persisted in 2022, with an increase to 36.3 cases per 100,000 inhabitants (78,057 cases; Figure 1B). As the global profile, the influence of the COVID-19 pandemic had a controversially shown negative impact on efforts to combat tuberculosis, deviating from the global expectation of reducing the number of cases. Based on the presented numbers, the COVID-19 pandemic resulted in setbacks for Brazil's national plans, which aimed to achieve < 10 cases per 100,000 inhabitants and restrict the annual number of deaths to < 230 by 2035.

The global and Brazilian decline in new cases during 2020 and 2021 may be attributed to the reduced number of diagnoses, a consequence of the substantial increase in SARS-CoV-2 infection cases and their severity. This surge placed an overwhelming burden on all health-related departments, diverting attention and resources away from tuberculosis diagnosis, thereby negatively impacting tuberculosis control (5). Contrastingly, despite its low recombination and mutation rate, it is noteworthy to emphasize the significant increase in new tuberculosis cases in 2022. This uptick is particularly surprising considering the period followed the peak of the pandemic, during which the adoption of sanitary measures was expected to potentially contribute to the reduction in the transmission of various airborne pathogens (6, 7).

The rising trajectory of epidemic tuberculosis cases prompts three crucial questions: (1) Why have the implemented sanitary measures not effectively curtailed tuberculosis transmission, particularly given that both pathogens are airborne? (2) Could the surge in new cases in 2022 be attributed to a heightened activation of latent tuberculosis? (3) Have there been any advancements in TB diagnoses that might explain the increase in numbers?

Concerning Question 1, we can postulate that the elevated TB numbers might be attributable to delays in notifications from 2020 and 2021, coupled with reductions in access to diagnosis and treatment. These factors could potentially influence the latest health reports of 2023, indicating a significant increase beyond the observed average rates when compared to trends before the COVID-19 pandemic (8). It is also a hypothesis that individuals could also have contracted the TB in their homes. Despite the adoption of masks, their use was predominantly in public spaces, and the primary transmission chain and the higher risk of TB involve familial and frequent contact with active tuberculosis cases, with an infectivity rate of 22% (9, 10). Indeed, it is estimated that an individual with active TB can infect an average of 10–15 people during the course of a year. In this regard, the use of masks may not have significantly influenced TB transmission, and new strategies should be studied and implemented to minimize damage to various healthcare sectors in future pandemics.

Discussion of Question 2 is paramount. The activation of latent tuberculosis is a dynamic process that can occur at any point, with the condition of the immune system playing a pivotal role in its reactivation. Notably, during the pandemic, a substantial number of individuals contracted COVID-19, and even in milder cases, this could potentially influence the activation of tuberculosis. It is essential to emphasize the complex interplay between the two pathogens, intensifying the severity of cases as a result of the immunologically sensitized state in the presence of both the virus and the bacteria. Due to their airborne transmission and the potential for significant damage to lung structures and cells, a cycle of negative feedback developed between these two pathogenic agents (11, 12). Moreover, the extended duration of the pandemic led to heightened psychological stresses, which may contribute to immunosuppression, further enhancing the likelihood of latent TB reactivation (13). In addition to psychosomatic factors, the SARS-CoV-2 infection itself is responsible for the excessive activation of the immune system, leading to an abundance of pro-inflammatory cytokine release, resulting in a “cytokine storm.” The presence of high levels and diversity of cytokines in the body triggers the hyperactivity of the immune system and the production of autoantibodies that can result in autoimmune diseases such as autoimmune hemolytic anemia, autoimmune thrombocytopenia, Guillain-Barré syndrome, vasculitis, multiple sclerosis, pro-thrombotic state, diffuse coagulopathy, and other autoinflammatory conditions, weakening the effectiveness of the immune system in maintaining chronic conditions such as latent tuberculosis, transitioning latent cases to active tuberculosis (14).

Regarding question 3, the increase in cases above 2019 observed in 2022 has been evidenced because of failures in containing new tuberculosis cases, lack of care, diagnoses, and notifications due to health system overload during the pandemic period, difficulties in the supply and transportation of medications experiencing disruptions due to flight cancellations and circulation restrictions. Additionally, quarantines and stay-at-home measures have increased the risk of TB transmission, especially among family members. Many of the strategies adopted during the COVID-19 pandemic may have been beneficial for containing SARS-CoV-2, but they have led to consequences that will still be listed in the coming years (15). There is another side of the coin that must be taken into consideration: despite the significant losses caused by the virus, numerous technological advancements have been developed with the intention of better facing the new challenges that would be encountered during the pandemic. Technological advancements were notable with various digital innovations, enabling remote learning, and the shift of major companies to the new “home office” format. In the realm of research and health, advancements can be highlighted in the search for faster and more effective diagnostic methods necessary for confirming COVID-19 cases and implementing necessary measures more rapidly. There have been developments in new platforms of machine learning for epidemiological studies and infectivity dynamics, as well as progress in vaccine development. In Brazil, innovations in the diagnosis of active tuberculosis cases began in 2020 with the implementation in the unified health system, through ordinance SCTIE/MS No. 34, of August 24, 2020, using a new methodology called “Automated Liquid Culture,” an automated technology for detecting mycobacteria and testing sensitivity to antimicrobials used in tuberculosis treatment simultaneously (16).

In Brazil, in 2022, Information Note No. 2/2022-CGDR/DCCI/SVS/MS detailed the adoption of new diagnostic tests known as Interferon-Gamma Release Assays (IGRA) for a more precise diagnosis of latent tuberculosis. The implementation process for latent tuberculosis diagnosis took place throughout 2022 and 2023, culminating in its permanent integration into the unified health system by 2024. This integration aims to facilitate frequent testing, particularly for children who have been in contact with active tuberculosis, HIV carriers, and patients undergoing immunosuppression, who are susceptible to developing active tuberculosis.

The full impact of the pandemic is yet to be comprehensively studied in the coming years. The return to desired case control parameters may occur gradually, offering a more coherent understanding of the global tuberculosis landscape. This understanding will inform the implementation of strategies aimed at reducing tuberculosis cases on a global scale. Detecting latent tuberculosis cases is crucial for both investigation and treatment of the infection before it becomes active. Alongside the establishment of a new network for latent testing, it's vital to highlight the significance of past health awareness campaigns in Brazil. These initiatives have played a pivotal role in disseminating knowledge about tuberculosis vaccination and promoting testing for both latent and active cases. Therefore, implementing public campaigns emphasizing the importance of tuberculosis vaccination and testing for suspected cases can significantly contribute to disease control.

The national scenario mirrors the global trend of recent years, indicating a notable rise in cases between 2017 and 2024, encompassing both pre- and post-pandemic periods (Figure 1C). To address the large number of tuberculosis cases in Brazil, it is essential to adopt a multifaceted approach that includes prevention, early diagnosis, effective treatment, and public awareness. In addition to the necessity of all these factors, another important point for recovery from the increase in cases is to improve treatment and adherence to treatment by ensuring the continuous and free supply of medications through the Unified Health System (SUS). Awareness and health education campaigns should be conducted across various media platforms and integrated into schools and communities to demystify the disease and reduce stigma, along with preventive measures such as promoting vaccination. The inclusion of epidemiological studies is necessary to better understand the patterns of transmission and risk factors in different regions of the country, where we can observe the heterogeneity in the distribution of cases (Figure 1C). Finally, it is crucial to ensure adequate and sustainable funding for tuberculosis control programs and to develop health policies, implementing these strategies in a coordinated and continuous manner.

The intricate interplay highlighted above emphasizes the necessity for a thorough understanding of the complex factors influencing the dynamics of tuberculosis within the context of the ongoing global health crisis. This prompts a critical analysis of the situation. The epidemiological data presented not only indicate the potential impacts of the COVID-19 pandemic on public health extending beyond the direct effects on its victims but also reveal the persistent challenge posed by tuberculosis. Indeed, despite being described by Robert Koch in 1882, this ancient disease remains uncontrolled and far from eradication.

The call for an efficient flow of communication and collaboration among governmental and international organizations, economic sectors, and the scientific community is not just a recommendation but a crucial imperative. These entities play a fundamental role in establishing a global system resilient to emerging pandemics, thereby mitigating potential damages that could significantly impact society during future outbreaks. The lessons drawn from the intersection of COVID-19 and tuberculosis underscore the importance of proactive and coordinated efforts to address global health crises.

## Author contributions

EF: Writing – original draft, Writing – review & editing, Conceptualization, Investigation, Methodology, Resources. MP: Writing – original draft, Writing – review & editing, Conceptualization, Investigation, Supervision. JA: Writing – review & editing, Supervision. PC: Writing – review & editing, Conceptualization, Investigation, Supervision.
